# Body fat, pericardial fat, liver fat and arterial health at age 10 years

**DOI:** 10.1111/ijpo.12926

**Published:** 2022-05-04

**Authors:** Giulietta S. Monasso, Susana Santos, Carolina C. V. Silva, Madelon L. Geurtsen, Edwin Oei, Romy Gaillard, Janine F. Felix, Vincent W. V. Jaddoe

**Affiliations:** ^1^ The Generation R Study Group, Erasmus MC University Medical Center Rotterdam Rotterdam the Netherlands; ^2^ Department of Pediatrics, Erasmus MC University Medical Center Rotterdam Rotterdam the Netherlands; ^3^ Department of Radiology and Nuclear Medicine, Erasmus MC University Medical Center Rotterdam Rotterdam the Netherlands

**Keywords:** cardiovascular disease, child, distensibility, epidemiology, intima‐media thickness, obesity

## Abstract

**Background:**

Body mass index is associated with carotid intima‐media thickness and distensibility in adults and children.

**Objective:**

To examine whether general and specific fat depots are associated with these markers of arterial health at school age.

**Methods:**

This cross‐sectional analysis was embedded in a population‐based prospective cohort study among 4708 children aged 10 years. Body, lean and fat mass index were estimated by dual‐energy X‐ray absorptiometry. Pericardial, visceral and liver fat were estimated by magnetic resonance imaging. Carotid intima‐media thickness and distensibility were measured by ultrasound.

**Results:**

A 1‐standard‐deviation‐score (SDS) higher body mass index was associated with higher carotid intima‐media thickness (0.06 SDS, 95% confidence interval [CI]: 0.03–0.08) and lower distensibility (−0.17 SDS, 95% CI: −0.20 to −0.14). These associations tended to be similar for lean mass index. A 1‐SDS higher fat mass index was associated with lower carotid intima‐media thickness (−0.08 SDS, 95% CI: −0.11 to −0.05) and lower distensibility (−0.10 SDS, 95% CI: −0.14 to −0.07). A 1‐SDS higher liver fat fraction was associated with lower carotid intima‐media thickness (−0.04 SDS, 95% CI: −0.08 to −0.00) and lower distensibility (−0.06 SDS, 95% CI: −0.10 to −0.03). We observed similar associations for visceral fat.

**Conclusions:**

At school age, lean and fat mass seem to be differentially related to carotid intima‐media thickness but not distensibility. Arterial development might be affected by lean mass, general and specific fat mass.

**Abbreviations**
CI: confidence interval
DXA: dual energy x‐ray absorptiometry
MRI: magnetic resonance imaging
SD: standard deviation
SDS: standard deviation score

## 1 INTRODUCTION

Higher body mass index seems to be a risk factor for higher carotid intima‐media thickness and lower carotid distensibility, two markers of arterial structure and function, respectively.^1^, ^2^, ^3^, ^4^, ^5^, ^6^ Body mass index is only a crude measure of adiposity, reflecting the sum of lean and fat mass without distinguishing between these components.^7^, ^8^, ^9^, ^10^ From an etiological point of view, it is important to identify which fat mass compartments relate to markers of arterial health. Previous observational studies in adults with obesity and healthy children suggest that subtle differences in carotid intima‐media thickness and distensibility may reflect pathological but also physiological changes in response to body size or growth.^6^, ^11^, ^12^ A large prospective cohort study reported positive associations of lean mass patterns across adolescence with carotid intima‐media thickness at age 17 years, whereas inverse associations were observed for fat mass patterns.^12^ Also, a cross‐sectional study among up to 838 children aged 9 years reported positive associations of lean mass index but not of fat mass index with carotid intima‐media thickness.^6^ The same study reported a stronger inverse association for lean mass index than fat mass index with carotid distensibility. It is not known if more specific compartments of fat mass, in particular, pericardial fat, visceral fat and liver fat, are associated with carotid intima‐media thickness and distensibility in healthy school‐age children. Pericardial fat, visceral fat and liver fat are systemically or locally acting ectopic fat depots that surround organs and blood vessels and that are associated with cardiovascular health.^7^, ^8^, ^9^, ^13^, ^14^, ^15^ Two small cross‐sectional studies among children and adolescents with obesity‐reported positive associations of epicardial fat or non‐alcoholic fatty liver disease with carotid intima‐media thickness.^16^, ^17^ Detailed population‐based studies on the associations of body composition with arterial health have not been performed yet.

In a population‐based prospective cohort study among 4728 children, we aimed to examine the associations of specific measures of body fat distribution, pericardial fat, visceral fat and liver fat mass, assessed by dual energy X‐ray absorptiometry and magnetic resonance imaging, with carotid intima‐media thickness and distensibility. Also, we examined whether these associations were independent of lean mass and fat mass.

## 2 METHODS

### 2.1 Design

This study was embedded in the Generation R Study, a population‐based prospective cohort study from foetal life onwards in the city of Rotterdam, the Netherlands.^18^ The Medical Ethical Committee of the Erasmus MC, University Medical Center Rotterdam approved the study (MEC 198.782/2001/31). Pregnant women living in Rotterdam and with an expected delivery date between April 2002 and January 2006 were eligible to participate. For the current study, data on lean mass and fat measures were available in 4708 singleton children with carotid intima‐media thickness or carotid distensibility measured at a median age of 9.7 years (95% range: 9.4, 10.5). For all participating children, we obtained written informed consent from their parents. A flowchart of the study population is shown in Figure S1.

### 2.2 Body fat measurements

At the age of 10 years, children were invited to our research facility.^18^ We measured height and weight without shoes or heavy clothing, from which we calculated body mass index (kg/m^2^) and subsequently sex‐ and age‐adjusted standard‐deviation‐scores (SDS), based on Dutch reference growth charts (Growth Analyser 4.0, Dutch Growth Research Foundation).^19^ We measured total body composition with a dual energy X‐ray absorptiometry (DXA) scanner (iDXA, Ge‐Lunar, 2008, Madison, WI, USA) using enCORE software version 12.6. We calculated the ratio of android and gynoid fat mass, which reflects the relation between fat mass in the abdominal (android) and hip (gynoid) regions.^20^ Further, we obtained pericardial fat mass, visceral fat mass and liver fat fraction from magnetic resonance imaging (MRI) scans, as described in detail previously and in the Supplemental Methods.^18^, ^21^ We previously created fat measures independent of height by adjusting the power to which height was elevated where needed to achieve complete independence.^21^, ^22^ Consequently, we defined lean mass index as (lean mass [kg] + bone mass [kg])/height (m^2^) and fat mass index as total fat mass (kg)/height (m^4^). We divided pericardial and visceral fat mass (kg) by height (m^3^) to obtain pericardial and visceral fat index, respectively.^22^ Android‐gynoid fat mass ratio was calculated as a marker of waist/hip fat distribution.^22^

### 2.3 Common carotid artery intima‐media thickness and distensibility

As described in detail previously, we measured common carotid artery intima‐media thickness and distensibility using the Logiq E9 (GE Medical Systems, Wauwatosa, WI, USA) device.^23^ Subsequent offline analyses were performed using the application Carotid Studio (Cardiovascular Suite [Quipu srl, Pisa, Italy]). Carotid intima‐media thickness was computed at the far wall as the average distance between lumen‐intima and media‐adventitia borders. The distensibility coefficient, or distensibility, was defined as the relative change in lumen area during systole for a given peripheral pressure change. We assessed peripheral blood pressure at the right brachial artery four times with the validated automatic sphygmomanometer Datascope Accutorr Plus (Paramus, NJ, USA).^24^ The lumen diameter of the carotid artery was computed as the average distance between the far and near media‐adventitia interfaces, for each frame of the acquired image sequence. Distension was calculated as the difference between the maximal (diastolic) and minimal (systolic) lumen diameter. The average distension and diameter values were used to compute the average carotid distensibility. We used the overall mean carotid intima‐media thickness (mm) and carotid distensibility (kPa^−1^ × 10^−3^) as outcomes of interest. In a reproducibility study, the interobserver and intraobserver intraclass correlation coefficients were >0.85.^23^

### 2.4 Covariates

We constructed a directed acyclic diagram (Figure S2). Potential covariates were selected based on previous literature and if adding them to the model led to a >10% change in effect estimate. We obtained information on maternal age, educational level and child ethnicity from questionnaires sent out during pregnancy and after birth, respectively. From midwife and hospital records, we obtained information on child sex.^18^

### 2.5 Statistical analysis

First, we performed a nonresponse analysis by comparing characteristics of children with and without carotid artery ultrasound data using Student's *t*‐tests, Mann–Whitney tests and Chi‐square tests. Second, we examined correlations of exposures and outcomes. Third, we used linear regression models to assess the cross‐sectional associations of body mass index, lean mass index, fat mass index, android‐gynoid ratio, pericardial fat index, visceral fat index and liver fat fraction with carotid intima‐media thickness and carotid distensibility at age 10 years. For each of these exposures, a separate model was constructed. To compare effect estimates, we analysed continuous exposures and outcomes in SDS, after natural log transformation carotid distensibility, which had a skewed distribution before transformation. The basic models were adjusted for sex and age at outcome measurement. The confounder models were additionally adjusted for child ethnicity and maternal age and education. Fourth, we performed several sensitivity analyses. We combined lean mass and fat mass index in one model to control for their effect on each other (mutually adjusted model). We only observed a statistically significant interaction between lean and fat mass for carotid distensibility (*p* = 0.05). We used conditional regression analyses to examine whether the associations of regional body fat measures with carotid intima‐media thickness and distensibility were independent of lean mass index and/or fat mass index. We constructed fat mass variables that are statistically independent of lean mass index and fat mass index, respectively, allowing simultaneous inclusion in multiple regression models. Details are presented in the Supplemental Methods. Last, we examined whether the associations were similar in the subgroup of children with complete information on all exposures and outcomes (main models). We used multiple imputations for covariates with missing values, using the Markov Chain Monte Carlo method. We created five datasets and report the pooled regression coefficients.^25^ We performed all statistical analyses using the Statistical Package of Social Sciences version 25.0 for Windows (SPSS IBM, Chicago, IL). As the exposures were correlated, we considered correction for multiple testing too strict and specify *p* < 0.05 and *p* < 0.01.

## 3 RESULTS

### 3.1 Subject characteristics

Tables 1 and S1 show all subject characteristics. The mean carotid intima‐media thickness was 0.46 (0.04 SD) mm, and the median carotid distensibility was 55.8 (95% range 37.1, 85.7) kPa^−1^ × 10^−3^. Compared with children with carotid artery measurements, those without outcomes on average were older when they visited the research centre and had less pericardial fat and liver fat (Table S2). Except for body mass index and lean mass index, which were positively correlated with carotid intima‐media thickness, all exposures were negatively correlated with both outcomes (Table S3).

**TABLE 1:** Subject characteristics (*n* = 4708)^a^

Maternal characteristics	
Age, mean (SD), years	30.9 (5.0)
Educational level	
No, primary, secondary, *n* (%)	2198 (50.9)
College or higher, *n* (%)	2121 (49.1)
Child characteristics at age 10 years	
Age at visit, median (95% range), years	9.7 (9.4, 10.5)
Sex	
Boy, *n* (%)	2346 (49.8)
Girl, *n* (%)	2362 (50.2)
Ethnicity	
European, *n* (%)	3107 (67.6)
Non‐European, *n* (%)	1492 (32.4)
Body mass index, median (95% range), kg/m^2^	17.0 (14.0, 24.8)
Lean mass index, mean (SD), kg/m^2^	12.5 (10.6, 14.9)
Fat mass index, mean (SD), kg/m^4^	2.2 (1.2, 5.0)
Android‐gynoid ratio, median (95% range), g	0.24 (0.15, 0.49)
Pericardial fat mass, median (95% range), g	10.7 (4.6, 22.7)
Visceral fat mass, median (95% range), g	358 (161, 980)
Liver fat fraction, median (95% range), %	2.0 (1.2, 5.3)
Common carotid artery intima‐media thickness, mean (SD), mm	0.46 (0.04)
Common carotid artery distensibility^b^, median (95% range), kPa^−1^ × 10^−3^	55.8 (37.1, 85.7)
Systolic blood pressure, mean (SD), mmHg	103 (8)
Diastolic blood pressure, mean (SD), mmHg	59 (6)
Non‐fasting glucose concentration, mean (SD), mmol/L	5.2 mmol/L (0.93)
Creatinine, mean (SD), μmol/L	47.0 (6.1)

^a^ Values are based on observed, not imputed data. We used multiple imputation for missing values: maternal age, *n* = 0; maternal educational level, *n* = 389; child age, *n* = 0; sex, *n* = 0; ethnicity, *n* = 109; body mass index, *n* = 11; lean mass index, *n* = 21; fat mass index, *n* = 21; android‐gynoid ratio, *n* = 10; pericardial fat mass, *n* = 2170; visceral fat mass, *n* = 2268.

^b^ Indicate values before natural‐log transformation.

### 3.2 Body mass, lean mass, fat mass, android‐gynoid ratio and arterial health

Compared with body mass index, the effect estimate of the association with carotid intima‐media thickness at age 10 years was larger for lean mass index (0.06 SDS [=0.0024], 95% confidence interval [CI]: 0.03–0.08 and 0.20 SDS [=0.008 mm], 95% CI: 0.17–0.23, respectively, per SDS; Table 2). Body and lean mass index showed similar inverse associations with carotid distensibility (−0.17 SDS [= −2.11 kPa^−1^ × 10^−3^], 95% CI: −0.20 to −0.14 and −0.19 SDS [= −2.36 kPa^−1^ × 10^−3^], 95% CI: −0.22 to −0.15, respectively, per SDS). Fat mass index and android‐gynoid ratio were both inversely associated with carotid intima‐media thickness (−0.08 SDS [= −0.0032 mm], 95% CI: −0.11 to −0.05 and −0.07 SDS [= −0.0028 mm], 95% CI: −0.10 to −0.04, respectively) and distensibility (−0.10 SDS [= −1.24 kPa^−1^ × 10^−3^], 95% CI: −0.14 to −0.07 and −0.10 SDS, 95% CI: −0.13 to −0.07, respectively). Basic models showed similar results (Table S4).

**TABLE 2:** Associations of body mass index, lean mass index, fat mass index and android‐gynoid ratio with carotid intima‐media thickness and carotid distensibility at age 10 years

	Carotid intima‐media thickness difference (95% confidence interval) *N* = 4708	Carotid distensibility difference (95% confidence interval) *N* = 4530
Body mass index, SDS (*n* = 4697)	0.06 (0.03, 0.08)**	−0.17 (−0.20, −0.14)**
Lean mass index, SDS (*n* = 4687)	0.20 (0.17, 0.23)**	−0.19 (−0.22, −0.15)**
Fat mass index, SDS (*n* = 4687)	−0.08 (−0.11, −0.05)**	−0.10 (−0.14, −0.07)**
Android‐gynoid ratio, SDS (*n* = 4698)	−0.07 (−0.10, −0.04)**	−0.10 (−0.13, −0.07)**

*Note*: Regression coefficients are linear regression coefficients based on standard‐deviation‐scores of carotid intima‐media thickness and carotid distensibility. Carotid distensibility was natural log‐transformed. Confounder models were adjusted for child sex and age at outcome measurement, child ethnicity, maternal age and education.

Abbreviation: SDS, standard‐deviation‐score.

* *p* < 0.05 (NA).

** *p* < 0.01.

### 3.3 Pericardial fat, visceral fat, liver fat and arterial health

Table 3 shows that pericardial fat index was not associated with carotid intima‐media thickness and distensibility. Visceral fat index was negatively associated with both carotid intima‐media thickness and distensibility (−0.07 SDS [= −0.0028 mm], 95% CI: −0.11 to −0.03 and −0.06 SDS [= −0.74 kPa^−1^ × 10^−3^], 95% CI: −0.10 to −0.02, per SDS, respectively). Also, liver fat fraction was negatively associated with carotid intima‐media thickness and distensibility (−0.04 SDS [= −0.0016 mm], 95% CI: −0.08 to −0.00 and −0.06 SDS [= −0.74 kPa^−1^ × 10^−3^], 95% CI: −0.10 to −0.03, respectively). Basic models showed similar results (Table S4).

**TABLE 3:** Associations of pericardial fat, visceral fat and liver fat with carotid intima‐media thickness and carotid distensibility at age 10 years

	Carotid intima‐media thickness difference (95% confidence interval) *N* = 4708	Carotid distensibility difference (95% confidence interval) *N* = 4530
Pericardial fat index, SDS (*n* = 2538)	−0.02 (−0.06, 0.02)	−0.04 (−0.08, 0.01)
Visceral fat index, SDS (*n* = 2440)	−0.07 (−0.11, −0.03)**	−0.06 (−0.10, −0.02)**
Liver fat fraction, SDS (*n* = 2768)	−0.04 (−0.08, −0.00)*	−0.06 (−0.10, −0.03)**

*Note*: Regression coefficients are linear regression coefficients based on standard‐deviation‐scores of carotid intima‐media thickness and carotid distensibility. Carotid distensibility was natural log‐transformed. Confounder models were adjusted for child sex and age at outcome measurement, child ethnicity, maternal age and education.

Abbreviation: SDS, standard‐deviation‐score.

* *p* < 0.05.

** *p* < 0.01.

### 3.4 Sensitivity analyses

In mutually adjusted models (Table S4), the associations of both lean and fat mass index with carotid intima‐media thickness had larger effect estimates compared with their respective confounder model. For carotid distensibility, the association with lean mas index was similar, but the association with fat mass index fully attenuated. Table S4 also shows that conditional on lean mass index, the associations of regional fat measures with carotid intima‐media thickness had larger effect estimates compared with their respective confounder model, whereas conditional on fat mass index, the associations of these fat measures with carotid intima‐media thickness fully attenuated. These findings suggest that associations of regional fat measures with carotid intima‐media thickness are partially determined by the positive association of lean mass index with carotid intima‐media thickness. Also, they suggest that when general fat mass is taken into account, regional fat mass accumulation is not associated with an additional risk of changes in carotid intima‐media thickness. For carotid distensibility, the observed associations partially or fully attenuated in the conditional analyses. This suggests that when either the lean mass or fat mass component of body mass index is taken into account, regional fat mass accumulation is not associated with an additional risk of changes in carotid distensibility. Results from the main models based on the subgroup with complete information on exposures and outcomes (*n* = 2135) were largely similar (Tables S5 and S6).

## 4 DISCUSSION

In this large population‐based prospective cohort study of healthy children aged 10 years, we observed that higher fat mass index, android‐gynoid ratio, visceral fat and liver fat fraction were all associated with both lower carotid intima‐media thickness and carotid distensibility. Conversely, higher body mass index and lean mass index were associated with higher carotid intima‐media thickness and lower distensibility.

We hypothesized that beyond body mass index, general fat mass, android‐gynoid ratio and pericardial fat, visceral fat and liver fat are associated with higher carotid intima‐media thickness and lower carotid distensibility at age 10 years. The identification of any associations might give clues about the aetiology of vascular disease.

A higher carotid intima‐media thickness may reflect early pathological atherosclerotic changes of the intima layer.^12^, ^26^ Additionally or alternatively, it may reflect physiological remodelling of the media layer in response to somatic growth.^6^, ^12^ We observed that in healthy children aged 10 years, body mass index and lean mass index were both positively associated with carotid intima‐media thickness. Not in line with our hypothesis, fat mass index, android‐gynoid ratio, visceral fat index and liver fat fraction were all inversely associated with carotid intima‐media thickness. Sensitivity analyses suggested that the associations of all fat measures with carotid intima‐media thickness were still confounded by the positive association between lean mass index and carotid intima‐media thickness.

Our findings, suggesting that lean mass index is the strongest determinant for carotid intima‐media thickness at age 10 years, are in line with two British cohort studies in children.^6^, ^12^ One of these, a prospective study among more than 4000 adolescents aged 17 years, reported that the positive association of body mass index with carotid intima‐media thickness was dependent on lean mass index.^12^ Our findings for pericardial fat are not in line with four cross‐sectional studies that assessed epicardial fat in children.^17^ These studies, among less than 240 children and adolescents predominantly with obesity, reported positive associations with carotid intima‐media thickness.^17^ Yet, epicardial and pericardial fat are correlated but seem anatomically, biochemically and clinically different, which makes them not directly comparable.^27^ Further, contrary to our findings for liver fat, a cross‐sectional study in Italy among 572 children aged 12 years of whom half had overweight, reported that non‐alcoholic fatty liver disease was positively associated with carotid intima‐media thickness.^16^ This was also observed in a study among 4143 elderly, generally with overweight, from the Multi‐Ethnic Study of Atherosclerosis.^28^ Our different findings compared with the previous studies might be explained by our healthy population with predominantly low liver fat fractions, or by age. Overall, it could be that in healthy children aged 10 years, rather than being a marker of subclinical atherosclerosis, higher carotid intima‐media thickness may reflect physiological remodelling of the media layer in response to lean mass growth. To confirm this, follow‐up studies are needed that link carotid intima‐media thickness at school‐age to health outcomes in later life.

Lower carotid distensibility is involved in the pathogenesis of cardiovascular disease.^29^ We observed that in healthy children aged 10 years, body mass index, lean mass index, fat mass index, android‐gynoid ratio, visceral fat index and liver fat fraction are all inversely associated with carotid distensibility. Sensitivity analyses showed that when either lean mass or fat mass was taken into account, android‐gynoid ratio, visceral fat index and liver fat fraction were not associated with an additional risk of changes in carotid distensibility. In line with our results, a British cross‐sectional study among 838 children aged 8 years reported stronger negative associations for lean mass than fat mass with carotid distensibility.^6^ Yet, the associations of fat and lean mass fully attenuated when adjusting for each other, whereas in our study, the association of lean mass with carotid distensibility remained. Research that links general and organ‐specific fat to arterial distensibility is limited. In 5770 elderly from the Multi‐Ethnic Study of Atherosclerosis, contrary to our findings, pericardial fat was inversely associated with carotid distensibility after adjusting for body mass index in men, although no association was observed in women.^30^ In line with our findings, one cross‐sectional study among 18 boys aged 12 years reported that aortic distensibility was negatively correlated with both abdominal visceral fat and liver fat.^31^ Conversely, the study among 4143 elderly from the Multi‐Ethnic Study of Atherosclerosis with a high prevalence of overweight and non‐alcoholic fatty liver disease reported a positive association between this fat measure and carotid distensibility.^28^ The different findings for liver fat in our study compared with the findings from the Multi‐Ethnic Study of Atherosclerosis may be explained by age and health status. To the best of our knowledge, we report for the first time that beyond body mass index, lean mass, general and abdominal fat mass, pericardial fat, visceral fat and liver fat are all negatively associated with carotid distensibility in healthy children aged 10 years.

Atherosclerosis and arterial stiffening are distinct processes, but they have common risk factors, often coexist and act synergistically.^29^, ^32^ The metabolic complications associated with obesity may mediate these associations and oxidative stress seems to be involved.^33^ Body mass index is the sum of lean and fat mass index but cannot distinguish between these components, which are both increased in individuals with obesity.^8^, ^9^, ^10^ Although body mass index is a common screening tool that has a high sensitivity to identify childhood adiposity, it has a moderate specificity.^34^ Fat mass and lean mass are both metabolically active, the latter more than the former. Higher fat mass and lean mass thus both increase oxygen demand. This requires higher blood flow and thus higher cardiac output, which increases blood pressure.^12^, ^35^ Higher blood pressure has been linked to higher intima‐media thickness and lower distensibility.^2^, ^32^, ^36^ Subtle differences in intima‐media thickness may reflect the equilibrium between the effects of pressure and flow on the carotids.^12^, ^37^ Higher lean mass does not change the intima‐media thickness/lumen ratio and thus local wall shear stress and tensile stress are kept constant.^12^, ^37^ Conversely, higher fat mass results in a reduced intima‐media thickness/lumen ratio and increased wall stress. This might increase fractional collagen engagement within the wall of the carotid artery and subsequently lead to progressive stiffening of the carotid artery. Thus, although both lean mass and fat mass are hypervolemic states, due to their opposite association with carotid intima‐media thickness, they may have different downstream effects.^12^ Distensibility is derived from the systolic–diastolic variations in arterial cross‐sectional area and local pulse pressure.^38^ Adaptive remodelling of the arterial wall in response to variation in blood pressure may explain subtle differences in carotid distensibility.^29^, ^32^, ^39^ Our findings emphasize that in healthy school‐age children, associations of body mass index with carotid intima‐media thickness and distensibility may partially reflect non‐pathological, physiological responses to hemodynamic changes.

Main strengths of this study are its population‐based prospective design and the availability of detailed DXA and MRI measurements and carotid artery ultrasound data. Some limitations need to be considered as well. First, we measured the exposures at one time point in childhood only and simultaneously with the outcomes. Thus, we cannot infer causality for the observational, cross‐sectional associations. Second, the majority of children in our population had a normal weight and thus our findings may not be observed in populations of children with obesity. Similarly, although we had a relatively large sample size, generalizability to other populations or subpopulations may be limited. Third, we calibrated carotid distensibility to brachial blood pressure, which will have shifted the calculated distensibility to higher values. Fourth, as in any observational study, we cannot exclude residual confounding in the observed associations, for example due to intrinsic endowments of the assessed exposures.

## 5 CONCLUSIONS

Our findings provide evidence that in healthy children aged 10 years, fat mass and lean mass seem to be differentially related to carotid intima‐media thickness but not carotid distensibility. Subtle differences in these arterial markers at school age may partially reflect an influence of general and organ fat mass accumulation or physiological adaptation to lean mass.

## FUNDING INFORMATION

The general design of the Generation R Study is made possible by financial support from the Erasmus MC, University Medical Centre Rotterdam, Erasmus University Rotterdam, the Netherlands Organization for Health Research and Development (ZonMw), the Netherlands Organization for Scientific Research (NWO), the Ministry of Health, Welfare and Sport and the Ministry of Youth and Families. Vincent W.V. Jaddoe received funding from the European Research Council (ERC‐2014‐CoG‐648 916). The project was supported by funding from the European Union's Horizon 2020 research and innovation program under grant agreements No 733206 (LifeCycle) and 874 739 (LongITools). Romy Gaillard received funding of the Dutch Heart Foundation (grant number 2017T013), the Dutch Diabetes Foundation (grant number 2017.81.002), the Netherlands Organization for Health Research and Development (NWO, ZonMW, grant number 543003109).

## CONFLICT OF INTEREST

No conflict of interest was declared.

## AUTHOR CONTRIBUTIONS

Vincent W.V. Jaddoe was responsible for conceptualization and design of this study. Giulietta S. Monasso analysed the data. Giulietta S. Monasso and Vincent W.V. Jaddoe interpreted the data. Giulietta S. Monasso wrote the original draft of the manuscript under the supervision of Vincent W.V. Jaddoe and Susana Santos, Carolina C.V. Silva, Edwin Oei, Romy Gaillard and Janine F. Felix were major contributors. All authors read and contributed to the preparation of the final manuscript. All authors read and approved the final manuscript.

## Supporting information


**Data S1**. Supporting information.
