# Supplementary material for: Body fat, pericardial fat, liver fat and arterial health at age 10 years
Source: Pediatr Obes. 2022 May 4;17(10):e12926. doi: 10.1111/ijpo.12926 (PMC9541379; doi:10.1111/ijpo.12926)
Supplement: Supplementary file 1 — Data S1. Supporting information. [file IJPO-17-e12926-s001.pdf]

Supporting Information

Body fat, pericardial fat, liver fat and arterial health at age 10 years

Giulietta S. Monasso[1,2], Susana Santos[1,2], Carolina C.V. Silva[1,2], Madelon L. Geurtsen[1,2], Edwin Oei, MD, PhD[3], Romy Gaillard[1,2], Janine F. Felix[1,2], Vincent W.V. Jaddoe[1,2]

1. The Generation R Study Group, Erasmus MC, University Medical Center Rotterdam, Rotterdam, the Netherlands
2. Department of Pediatrics, Erasmus MC, University Medical Center Rotterdam, Rotterdam, the Netherlands
3. Department of Radiology & Nuclear Medicine

Corresponding Author

Vincent W.V. Jaddoe, The Generation R Study Group (Na 29 – 08). Erasmus MC, University Medical Center Rotterdam, PO Box 2040, 3000 CA Rotterdam, the Netherlands. Phone: +31 10 7043405, Fax: +31 10 7044645, Email: v.jaddoe@erasmusmc.nl

Content

- Supplemental Methods
- Figure S1. Flow chart of the study population
- Figure S2. Directed acyclic graph
- Table S1. Subject characteristics after imputation for covariates (n=4708)
- Table S2. Non-response analysis (n=5312)
- Table S3. Correlation matrix of exposures and outcomes
- Table S4. Associations of body mass, lean mass, fat mass, pericardial fat, visceral fat and liver fat with carotid intima-media thickness and carotid distensibility at age 10 years
- Table S5. Associations of body mass index, lean mass index, fat mass index and android-gynoid ratio with carotid intima-media thickness and carotid distensibility at age 10 years among children with information on all exposures and outcomes (n=2135)
- Table S6. Associations of pericardial fat, visceral fat and liver fat with carotid intima-media thickness and carotid distensibility at age 10 years among children with information on all exposures and outcomes (n=2135)

SUPPLEMENTAL METHODS

Assessment of pericardial fat, visceral fat and liver fat

At age 10 years, we obtained visceral, pericardial and liver fat from magnetic resonance imaging (MRI) scans. Children were scanned using a 3.0 Tesla MRI (Discovery MR 750w, GE Healthcare, Milwaukee, WI, USA). Pericardial fat imaging in short axis orientation was performed using an electrocardiogram-triggered black-blood-prepared thin slice single shot fast spin echo acquisition with multi-breath-hold approach. An axial 3-point Dixon acquisition for fat and water separation (IDEAL IQ) was used for liver fat imaging.[1] An axial abdominal scan from lower liver to pelvis and a coronal scan centered at the head of the femurs were performed with a 2-point DIXON acquisition (LavaFlex). The obtained fat scans were subsequently analyzed by the Precision Image Analysis company (PIA, Kirkland, WA, USA), using the sliceOmatic (TomoVision, Magog, Canada) software package. Extraneous structures and any image artifacts were removed manually.[2] Pericardial fat included both epicardial and paracardial fat directly attached to the pericardium, ranging from the apex to the left ventricular outflow tract. Total visceral fat volume ranged from the dome of the liver to the superior part of the femoral head. Fat mass was obtained by multiplying the total volumes by the specific gravity of adipose tissue, 0.9 g/mL. Liver fat fraction was determined by taking 4 samples of at least 4 cm2 from the central portion of the hepatic volume. Subsequently, the mean signal intensities were averaged to generate overall mean liver fat fraction estimation.

Conditional regression analyses

Body mass index is only a crude measure of adiposity, reflecting the sum of lean and fat mass without distinguishing between these components.[8-11] Also, the associations of android-gynoid ratio, pericardial fat, visceral fat and liver fat with carotid intima-media thickness and carotid distensibility may not be statistically independent from lean mass index and/or fat mass index. To further examine this, we performed conditional regression modelling as sensitivity analyses. First, we built conditional

models for each of the regional fat measures using linear regression analyses. In the lean mass conditional models, we obtained the standardized residual for each regional fat measure from the regression of the respective fat measure on lean mass index. These standard residuals are completely uncorrelated with lean mass index. We used the same approach for fat mass index: in the fat mass conditional models, we obtained the standardized residual for each regional fat measure from the regression of the respective fat measure on fat mass index. These standard residuals are completely uncorrelated with fat mass index.[3,4] Thus, for each regional fat measure, we constructed one new variable independent of lean mass, and one new variable independent of fat mass. Second, for each of the two independent variables per regional fat measure, we used conventional linear regression analyses (confounder models) to assess their associations with carotid intima-media thickness. The obtained results were thus independent of lean mass and fat mass, respectively. Third, in a similar manner we assessed the associations of the two standard residuals per regional fat measure with carotid distensibility, independent of lean mass and fat mass, respectively. Fourth, for each regional fat measure, we were able to compare the results that we obtained from the confounder model to the results that were independent of lean and fat mass, respectively (**Table S4**).

References

1. Reeder SB, Cruite I, Hamilton G, Sirlin CB. Quantitative Assessment of Liver Fat with Magnetic Resonance Imaging and Spectroscopy. J Magn Reson Imaging. 2011;34(4):729-749.

2. Hu HH, Nayak KS, Goran MI. Assessment of abdominal adipose tissue and organ fat content by magnetic resonance imaging. Obes Rev. 2011;12(5):e504-515.

3. Keijzer-Veen MG, Euser AM, van Montfoort N, Dekker FW, Vandenbroucke JP, Van Houwelingen HC. A regression model with unexplained residuals was preferred in the analysis of the fetal origins of adult diseases hypothesis. J Clin Epidemiol. 2005 Dec;58(12):1320-4.

4. Gishti O, Gaillard R, Durmus B, et al. BMI, total and abdominal fat distribution, and cardiovascular risk factors in school-age children. Pediatr Res. 2015 May;77(5):710-8.

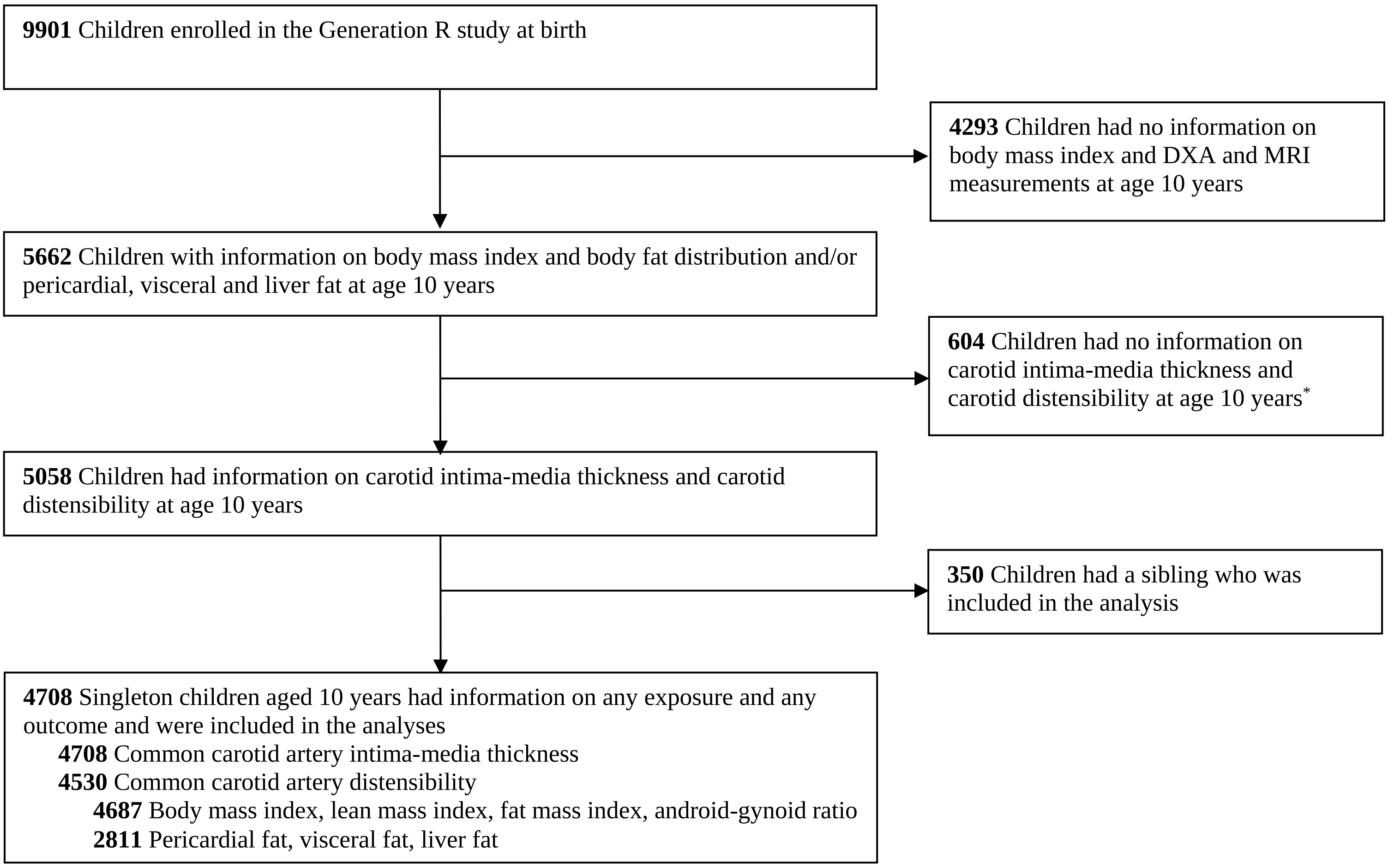

Figure S1. Flow chart of the study population

* The non-response analysis compared included children to the 604 children who had information on any exposure but were not included in the analysis because they had no information on common carotid artery intima-media thickness or distensibility available.

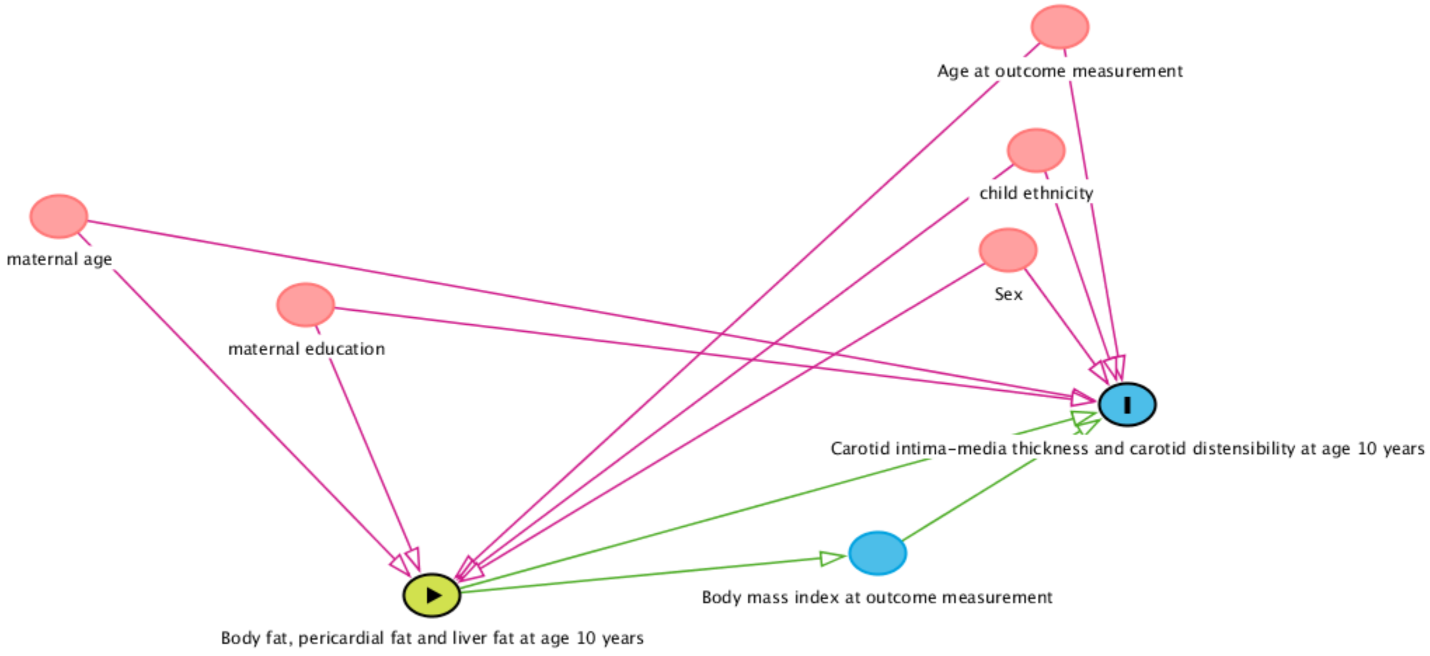

Figure S2. Directed acyclic graph

Directed acyclic graph represents assumptions of a causal relationship between the exposures and outcomes. Exposures included body mass index, lean mass index, fat mass index, android-gynoid ratio, visceral fat index, pericardial fat index and liver fat fraction. The other variables are considered confounders or potential mediators.

Table S1. Subject characteristics after imputation for covariates (n=4708)*

**Maternal characteristics**	
Age, mean (SD), y	30.9 (5.0)
Educational level	
No, primary, secondary, n (%)	2440 (50.8)
College or higher, n (%)	2268 (48.2)
**Child characteristics at age 10 years**	
Age at visit, median (95% range), y	9.7 (9.4, 10.5)
Sex	
Boy, n (%)	2346 (49.8)
Girl, n (%)	2362 (50.2)
Ethnicity	
European, n (%)	3172 (67.4)
Non-European, n (%)	1536 (32.6)
Body mass index, median (95% range), kg/m^2	17.0 (14.0, 24.8)
Lean mass index, mean (SD), kg/m^2	12.5 (10.6, 14.9)
Fat mass index, mean (SD), kg/m^4	2.2 (1.2, 5.0)
Android-gynoid ratio, median (95% range), g	0.24 (0.15, 0.49)
Pericardial fat mass, median (95% range), g	10.7 (4.6, 22.7)
Visceral fat mass, median (95% range), g	358 (161, 980)
Liver fat fraction, median (95% range), %	2.0 (1.2, 5.3)
Common carotid artery intima-media thickness, mean (SD), mm	0.46 (0.04)
Common carotid artery distensibility†, median (95% range), $kPa^{-1}*10^{-3}$	55.8 (37.1, 85.7)
Mean arterial pressure	74 (6)

* Exposures and outcomes were not imputed. Missing values: maternal age, n=0; maternal educational level, n=389; child age, n=0; sex, n=0; ethnicity, n=109; body mass index, n=11; lean mass index, n=21; fat mass index, n=21; android-gynoid ratio, n=10; pericardial fat mass, n=2170; visceral fat mass, n=2268; mean arterial pressure, n=30.

† Indicate values before natural-log transformation.

Table S2. Non-response analysis (n=5312)*

	Included (n=4708)	Not included (n=604)	P-value
**Maternal characteristics**			
Age, mean (SD), y	30.9 (5.0)	30.9 (5.1)	0.99
Educational level			0.26
No, primary, secondary, n. (%)	2198 (50.9)	266 (48.4)	
College or higher, n (%)	2121 (49.1)	284 (51.6)	
**Child characteristics at age 10 years**			
Age at visit, median (95% range), y	9.7 (9.4, 10.5)	9.8 (9.4, 11.2)	<0.001
Sex			0.82
Boy, n (%)	2346 (49.8)	304 (50.3)	
Girl, n (%)	2362 (50.2)	300 (49.7)	
Ethnicity			0.08
European, n (%)	3107 (67.6)	373 (64.0)	
Non-European, n (%)	1492 (32.4)	210 (36.0)	
Body mass index, median (95% range), kg/m^2	17.0 (14.0, 24.8)	16.9 (14.1, 25.1)	0.57
Lean mass index, mean (SD), kg/m^2	12.5 (10.6, 14.9)	12.6 (10.7, 14.9)	0.08
Fat mass index, mean (SD), kg/m^4	2.2 (1.2, 5.0)	2.2 (1.2, 5.2)	0.56
Android-gynoid ratio, median (95% range), g	0.24 (0.15, 0.49)	0.24 (0.15, 0.50)	0.77
Pericardial fat mass, median (95% range), g	10.7 (4.6, 22.7)	10.1 (4.5, 23.8)	0.01
Visceral fat mass, median (95% range), g	358 (161, 980)	340 (160, 1139)	0.07
Liver fat fraction, median (95% range), %	2.0 (1.2, 5.3)	1.9 (1.2, 5.6)	0.001
Mean arterial pressure	74 (6)	72 (7)	<0.001

* Values are based on observed, not imputed data. The non-response analysis compared included children to the 604 children who had information on any exposure but were not included in the analysis because they had no information on common carotid artery intima-media thickness or distensibility available. Differences in subject characteristics between included children and those not included were calculated using Student's t-tests, Mann-Whitney tests and Chi-square tests

Table S3. Correlation matrix of exposures and outcomes*

	Body mass index	Lean mass index	Fat mass index	Android/gynoid ratio	Pericardial fat index	Visceral fat index	Liver fat fraction	Carotid intima-media thickness	Carotid distensibility
Body mass index	1.000	**0.678**‡	**0.832**‡	**0.728**‡	**0.325**‡	**0.622**‡	**0.437**‡	**0.047**‡	**-0.165**‡
Lean mass index		1.000	**0.274**‡	**0.275**‡	**0.153**‡	**0.202**‡	**0.171**‡	**0.211**‡	**-0.207**‡
Fat mass index			1.000	**0.779**‡	**0.363**‡	**0.717**‡	**0.441**‡	**-0.09**‡	**-0.065**‡
Android/gynoid ratio				1.000	**0.332**‡	**0.684**‡	**0.438**‡	**-0.063**‡	**-0.097**‡
Pericardial fat index					1.000	**0.491**‡	**0.181**‡	-0.019	-0.030
Visceral fat index						1.000	**0.422**‡	**-0.084**‡	**-0.049†**
Liver fat fraction							1.000	-0.022	**-0.077**‡
Carotid intima-media thickness								1.000	**-0.171**‡
Carotid distensibility									1.000

* Values are Pearson correlation coefficients.
†P-value <0.05; ‡P-value <0.01

Table S4. Associations of body mass, lean mass, fat mass, pericardial fat, visceral fat and liver fat with carotid intima-media thickness and carotid distensibility at age 10 years

	**Carotid intima-media thickness Difference (95% confidence interval) N=4708**	**Carotid distensibility Difference (95% confidence interval) N=4530**
Body mass index, SDS (n=4697)		
Basic model	**0.06 (0.03. 0.08)†**	**-0.16 (-0.19, -0.14)†**
Confounder model	**0.06 (0.03, 0.08)†**	**-0.17 (-0.20, -0.14)†**
Lean mass index, SDS (n=4687)		
Basic model	**0.20 (0.17, 0.23)†**	**-0.19 (-0.22, -0.16)†**
Confounder model	**0.20 (0.17, 0.23)†**	**-0.19 (-0.22, -0.15)†**
Mutually adjusted model ‡	**0.27 (0.24, 0.31)†**	**-0.17 (-0.21, -0.14)†**
Fat mass index, SDS (n=4687)		
Basic model	**-0.06 (-0.09, -0.03)†**	**-0.10 (-0.13, -0.07)†**
Confounder model	**-0.08 (-0.11, -0.05)†**	**-0.10 (-0.14, -0.07)†**
Mutually adjusted model ‡	**-0.19 (-0.23, -0.16)†**	-0.03 (-0.06, 0.01)
Android-gynoid ratio, SDS (n=4698)		
Basic model	**-0.06 (-0.09, -0.03)†**	**-0.11 (-0.13, -0.08)†**
Confounder model	**-0.07 (-0.10, -0.04)†**	**-0.10 (-0.13, -0.07)†**
Lean mass index model ǁ	**-0.13 (-0.16, -0.10)†**	**-0.05 (-0.08, -0.02)†**
Fat mass index model #	-0.01 (-0.04, 0.02)	**-0.05 (-0.08, -0.02)†**
Pericardial Fat Index, SDS (n=2538)		
Basic model	-0.02 (-0.06, 0.02)	**-0.04 (-0.08, 0.00)***
Confounder model	-0.02 (-0.06, 0.02)	-0.04 (-0.08, 0.01)
Lean mass index model §	**-0.05 (-0.09, -0.01)***	-0.01 (-0.05, 0.03)
Fat mass index model ǁ	0.00 (-0.04, 0.04)	-0.00 (-0.04, 0.04)
Visceral Fat Index, SDS (n=2440)		
Basic model	**-0.07 (-0.11, -0.03)†**	**-0.07 (-0.11, -0.03)†**
Confounder model	**-0.07 (-0.11, -0.03)†**	**-0.06 (-0.10, -0.02)†**
Lean mass index model §	**-0.12 (-0.16, -0.08)†**	-0.02 (-0.06, 0.02)
Fat mass index model ǁ	-0.04 (-0.08, 0.00)	0.02 (-0.03, 0.06)
Liver fat fraction, SDS		
Basic model	-0.04 (-0.08, 0.00)	**-0.07 (-0.11, -0.03)†**
Confounder model (n=2768)	**-0.04 (-0.08, -0.00)***	**-0.06 (-0.10, -0.03)†**
Lean mass index model §	**-0.08 (-0.11, -0.04)***	-0.04 (-0.07, 0.00)
Fat mass index model ǁ	-0.02 (-0.06, 0.02)	-0.03 (-0.07, 0.01)

SDS: standard-deviation-score
Regression coefficients are linear regression coefficients based on standard-deviation-scores of carotid intima-media thickness and carotid distensibility. Carotid distensibility was natural log-transformed. Models were adjusted for child sex and age at outcome measurement. Confounder models were additionally adjusted for child ethnicity, maternal age and education. *P<0.05, †P<.01.
‡ In the mutually adjusted model we additionally adjusted the confounder model for fat mass and lean mass index, respectively.
§ Based on conditional regression analyses in which the respective fat measure was regressed on lean mass index to create fat measures independent of lean mass index. These models were not run for body mass index, as it is defined as the sum of lean and fat mass index.
|| Based on conditional regression analyses in which the respective fat measure was regressed on fat mass index to create fat measures independent of fat mass index. These models were not run for body mass index, as it is defined as the sum of lean and fat mass index.

Table S5. Associations of body mass index, lean mass index, fat mass index and android-gynoid ratio with carotid intima-media thickness and carotid distensibility at age 10 years among children with information on all exposures and outcomes (n=2135)

	Carotid intima-media thickness Difference (95% confidence interval)	Carotid distensibility Difference (95% confidence interval)
Body mass index, SDS (n=4697)	**0.09 (0.05, 0.14)**†	**-0.16 (-0.20, -0.12)**†
Lean mass index, SDS (n=4687)	**0.24 (0.19, 0.28)**†	**-0.20 (-0.24, -0.15)**†
Fat mass index, SDS (n=4687)	**-0.08 (-0.13, -0.03)***	**-0.10 (-0.15, -0.06)**†
Android-gynoid ratio, SDS (n=4698)	-0.04 (-0.08, -0.01)	**-0.10 (-0.14, -0.05)**†

SDS: standard-deviation-score. Regression coefficients are linear regression coefficients based on standard-deviation-scores of carotid intima-media thickness and carotid distensibility. Carotid distensibility was natural log-transformed. Confounder models were adjusted for child sex and age at outcome measurement, child ethnicity, maternal age and education. *P<0.05 (NA), †P<0.01.

Table S6. Associations of pericardial fat, visceral fat and liver fat with carotid intima-media thickness and carotid distensibility at age 10 years among children with information on all exposures and outcomes (n=2135)

	Carotid intima-media thickness Difference (95% confidence interval)	Carotid distensibility Difference (95% confidence interval)
Pericardial Fat Index, SDS (n=2538)	-0.01 (-0.06, 0.03)	-0.03 (-0.07, 0.01)
Visceral Fat Index, SDS (n=2440)	**-0.08 (-0.12, -0.03)†**	**-0.05 (-0.09, -0.01)***
Liver fat fraction, SDS (n=2768)	**-0.04 (-0.08, 0.01)**	**-0.07 (-0.12, -0.03)†**

SDS: standard-deviation-score. Regression coefficients are linear regression coefficients based on standard-deviation-scores of carotid intima-media thickness and carotid distensibility. Carotid distensibility was natural log-transformed. Confounder models were adjusted for child sex and age at outcome measurement, child ethnicity, maternal age and education. *P<0.05, †P<0.01.
